# Occurrence of antibiotic resistance in bacteria isolated from seawater organisms caught in Campania Region: preliminary study

**DOI:** 10.1186/1746-6148-10-161

**Published:** 2014-07-15

**Authors:** Giorgio Smaldone, Raffaele Marrone, Silvia Cappiello, Giuseppe A Martin, Gaetano Oliva, Maria L Cortesi, Aniello Anastasio

**Affiliations:** 1 Departement of Veterinary Medicine and Animal Production, University of Naples "Federico II", via F. Delpino 1, 80137 Naples, NA, Italy; 2 Departement of Veterinary Medicine and Animal Production, Veterinary pharmacovigilance center, Campania region, via F. Delpino 1, 80137 Naples, NA, Italy

**Keywords:** Antibiotic resistance, *Vibrio* strains, Fish, Antibiotic residues

## Abstract

**Background:**

Environmental contamination by pharmaceuticals is a public health concern: drugs administered to humans and animals are excreted with urine or faeces and attend the sewage treatment. The main consequences of use and abuse of antibiotics is the development and diffusion of antibiotic resistance that has become a serious global problem. Aim of the study is to evaluate the presence of antimicrobial residues and to assess the antimicrobial resistance in bacteria species isolated from different wild caught seawater fish and fishery products.

**Results:**

Three antibiotic substances (Oxytetracicline, Sulfamethoxazole and Trimethoprim) were detected (by screening and confirmatory methods) in *Octopus vulgaris*, *Sepia officinalis* and *Thais haemastoma*. All *Vibrio* strains isolated from fish were resistant to Vancomycin (VA) and Penicillin (P). In *Vibrio alginolyticus,* isolated in *Octopus vulgaris*, a resistance against 9 antibiotics was noted.

**Conclusions:**

Wild caught seawater fish collected in Gulf of Salerno (Campania Region), especially in marine areas including mouths of streams, were contaminated with antibiotic-resistant bacteria strains and that they might play an important role in the spread of antibiotic-resistance.

## Background

Environmental contamination by pharmaceuticals is a public health concern. Medical substances may roughly be divided into medical substances used by human or veterinary medicine. The veterinary drugs may further be subdivided into substances used as growth promoters for livestock production, therapeutics in livestock productions, coccidiostatic used for poultry production, therapeutics for treatment of livestock on fields or as feed additives in fish farms.

Drugs administered to humans and animals are excreted with urine or faeces [1] and attend the sewage treatment plant [2]; successively if substances are hydrophilic or are metabolized to a more hydrophilic form of the parent lipophilic substance, will pass the waste water treatment plant and end up in the receiving waters where they may be are present at very low concentrations; it is important noted that several substances could stimulate a response in humans and animals also at low doses with a very specific target [3]. A recent study showed that a mixture of drugs at the concentrations actually found in the aquatic environment of some Italian areas is able to exert toxic effects on the proliferation of human and zebra fish (*Danio rerio*) cells cultures [4]. The main consequences of use and abuse of antibiotics is the development and diffusion of antibiotic resistance that represent a public health problem, with obvious consequences in human and veterinary medicine, since it affects animal therapy and food safety [5,6]. World wide there is growing concern about the increased prevalence of antibiotic resistance: the growing alarm related to the spreading of the resistance of antibiotics considered of first choice in the treatment of specific human infections prompted measures for antimicrobial resistance surveillance of bacteria circulating in humans, animals and food products. Aim of this study was to evaluate the presence of antimicrobial substances and to assess the antimicrobial resistance in bacteria species isolated from wild caught seawater fish and fishery products caught in Tyrrenian sea along the coast of Campania region (southern Italy). The antibiotics tested were Teicoplanin (TEC), Cephalexin (CN), Penicillin (P), Oxacillin (OX), Amoxicillin/Clavulanic Acid (AMC), Cefotaxime (CTX), Vancomycin (VA), Sulfamethoxazole (SXT), Rifampicin (RD), Cefoxitin (FOX), Plaritromicin (PRL), Ciprofloxacin (CIP), Chloramphenicol (C), Tobramicin (TOB), Tetracycline (TE), Tigecycline (TGC), Linezolid (LZD) and Fosfomycin (FOS).

## Results and discussion

### Microbial analysis and antibiogram

The microbial species isolated were *Vibrio alginolyticus (Va), Vibrio parahaemolyticus (Vp)*, *Shewanella putrefaciens (Sp)* and *Acromonas spp. (Ac)* (Table 1); *Vp* is a bacteria naturally present in marine and estuarine aquatic environments and is part of the natural flora of fish and bivalve mollusks. *Va,* isolated from *Sepia officinalis* and *Trachurus trachurus* samples, is frequently detected from fin fish, shellfish, seawater, and sediment [7]. It has not been not widely recognized as a fish pathogen. *Sp* a microorganism common in marine environments as saprophytic is one of the major causes of spoilage of fish and fishery products [8]; Water bacteria might be indigenous to aquatic environments, or exogenous, transiently and occasionally present in the water as a result of shedding from animal, vegetal, or soil surfaces [9].

**Table 1:** Microbial species isolated in seawater fish and fishery products

**Microbial species**	**Samples**									
	***S. officinalis***	***M. cephalus***	***T. trachurus***	***S. scrofa***	***O. vulgaris***	***L. viridid***	***G. cobitis***	***S. umbra***	***D. sargus***	***T. haemastoma***
***V. cholerae***	***-***	***-***	***-***	***-***	***-***	***-***	***-***	***-***	***-***	***-***
***V. parahaemolyticus***	***+***	***-***	***+***	***+***	***-***	***-***	***-***	***-***	***+***	***-***
***V. vulnificus***	***-***	***-***	***-***	***-***	***-***	***-***	***-***	***-***	***-***	***-***
***V. fluvialis***	***-***	***-***	***-***	***-***	***-***	***-***	***-***	***-***	***-***	***-***
***V. mimicus***	***-***	***-***	***-***	***-***	***-***	***-***	***-***	***-***	***-***	***-***
***Other spp.***	*Va*	*Va*	*Va*	*Va*	*Va*	*Va*	*-*	*Va*	*Va*	*-*
***Other generes***	*Sp*	*-*	*-*	*Sp;Ac*	*Sp*	*-*	*Sp*	*-*	*-*	*Sp*

Different microbial species were isolated: *Vibrio parahaemolyticus (Vp)* was isolated in *Sepia officinalis, Trachurus trachurus, Scorpaena scrofa* and *Diplodus sargus*; *Vibrio alginolyticus (Va)* was isolated in all samples except in *Gobius cobitis* and *Thais haemastoma*; *Shewanella putrefaciens (Sp)* was isolated in cuttlefish, *Scorpaena scrofa*, *Octopus vulgaris, Gobius cobitis* and *Thais haemastoma*; *Acromonas spp. (Ac)* was isolated only in *Scorpaena scrofa.*

The frequency of antibiotic resistance among microbial strains isolated was shown in Table 2 and Table 3. Although only 7 species of fish and 3 species of fishery products were studied to determine the incidence of antibiotic resistance, all the strains isolated were resistant to one or more of the antibiotics tested; the frequency of resistance varied from 16.6% to 50% in different samples; 69,45% of the microbial strains isolates showed resistance to more than 4 molecules tested. *Va* showed antimicrobial resistance against 9 antibiotics, *Vp* against 4 antibiotics, *Sp* against 6 antibiotics and *Ac* against 4 antibiotics tested. Accordinto to Martinez [9], more than 90% of bacterial strains originated in seawater are resistant to more than one antibiotic. Multiple antibiotic resistance has been reported in a wide range of human pathogenic or opportunistic bacteria such as *Campylobacter spp*. [10], *Klebsiella pneumoniae*[11], *Salmonella sp.*[12], *Pseudomonas aeruginosa*[13], *E. coli*[14] and also in fish pathogens [15]. In all bacterial strains resistance against TEC and VA, drugs belonging to the class of glycopeptides having similar mechanisms of action on bacterial cell wall synthesis, and against P and OX, drugs belonging to the class of β-lactam antibiotics, was observed. Spectra of activity of TEC and VA are limited to Gram–positive bacteria including methicillin–resistant strains of *S. aureus* and *S. epidermidis* and for this reason the resistant Gram–negative bacteria isolates could be not sensitive to mechanism of action of these molecules. VA has a shorter half–life than TEC and requires multiple dosing to maintain adequate serum levels. In contrast, the pharmacokinetics of TEC allow for once–daily dosing and it is a drug associated with a lower incidence of nephrotoxicity or ototoxicity. For these reasons TEC is more cost–effective and its role in hospitals is likely to increase. Resistance to VA could be related not only to the use of VA in human medicine but also to a cross-resistance due to the use of *Avoparcin*, a glycopeptides utilized to improve performance in poultry flocks [16], which are present in the area near to sampling zone. Resistance against P and OX could be related to the large use of β-lactam antibiotics in human and veterinary medicine. The sensitivity against C detected in all bacterial strains coupled with the absence of C residues in fish sampled, confirm the limited administration to humans and the compliance on the use of the drug for food producing animals, banned since 1995. However, according Kerry *et al*. [17], it is important to underline that resistance phenomena are not systematically correlated with the presence of the corresponding drugs.

**Table 2:** Frequency of antibiotic resistance among the bacteria isolated

	**Antibiotics tested**																	
**Samples**	**TEC**	**CN**	**P**	**OX**	**AMC**	**CTX**	**VA**	**SXT**	**RD**	**FOX**	**PRL**	**CIP**	**C**	**TOB**	**TE**	**TGC**	**LZD**	**FOS**
***Va *** **isolated from *S. umbra***	R	S	R	R	R	S	R	I	S	I	R	S	S	I	S	I	S	I
***Va *** **isolated from *S. umbra***	R	I	R	R	R	S	R	S	S	I	S	S	S	I	S	S	S	S
***Va *** **isolated from *S. officinalis***	R	R	R	R	R	R	R	I	I	R	R	S	S	I	S	I	S	S
***Va *** **isolated from *S. officinalis***	S	S	R	R	R	S	R	S	S	I	R	S	S	I	R	S	S	S
***Va *** **isolated from *S. officinalis***	R	S	R	S	S	S	R	S	S	I	S	S	S	S	S	S	S	S
***Va *** **isolated from *T. trachurus***	R	I	R	R	R	S	R	I	S	I	I	S	S	S	S	S	S	S
***Va *** **isolated from *T. trachurus***	R	I	R	R	R	R	R	R	I	I	R	S	S	S	S	S	S	S
***Va *** **isolated from *T. trachurus***	R	I	R	R	R	R	R	I	I	I	R	S	I	I	S	S	S	S
***Va *** **isolated from *T. trachurus***	R	S	R	R	R	I	R	I	I	I	R	S	S	I	S	S	S	I
***Va *** **isolated from *T. trachurus***	R	I	R	R	I	S	R	S	S	I	S	S	S	I	S	S	S	S
***Va *** **isolated from *O. vulgaris***	S	S	R	R	R	S	R	S	S	I	R	S	S	I	R	S	S	S
***Va *** **isolated from *O. vulgaris***	R	I	R	R	S	S	R	S	S	I	R	S	S	I	S	S	S	S
***Va *** **isolated from *O. vulgaris***	R	I	R	R	S	S	R	S	S	I	R	S	S	I	S	S	S	S
***Va *** **isolated from *M. cephalus***	R	S	R	R	R	S	R	I	S	S	S	S	S	I	S	S	S	S
***Va *** **isolated from *M. cephalus***	R	I	R	R	S	S	R	S	S	I	R	S	S	I	S	S	S	S
***Va *** **isolated from *M. cephalus***	R	I	R	R	R	S	R	S	S	I	S	S	S	I	S	S	S	S
***Va *** **isolated from *M. cephalus***	R	S	R	R	R	S	R	I	S	I	R	S	S	I	S	I	S	I
***Va *** **isolated from *L. viridis***	R	I	R	R	I	S	R	S	S	I	S	S	S	I	S	S	S	S
***Va *** **isolated from *L. viridis***	R	S	R	R	R	S	R	I	S	S	S	S	S	I	S	S	S	S
***Va *** **isolated from *D. sargus***	R	I	R	R	R	S	R	I	S	I	I	S	S	I	S	S	S	S
***Va *** **isolated from *D. sargus***	R	I	R	R	R	S	R	I	S	I	I	S	S	I	S	S	S	S
***Va *** **isolated from *D. sargus***	R	I	R	R	R	S	R	I	S	I	I	S	S	I	S	S	S	S

Teicoplanin (TEC), Cephalexin (CN), Penicillin (P), Oxacillin (OX), Amoxicillin/Clavulanic Acid (AMC), Cefotaxime (CTX), Vancomycin (VA), Sulfamethoxazole (SXT), Rifampicin (RD), Cefoxitin (FOX), Plaritromicin (PRL), Ciprofloxacin (CIP), Chloramphenicol (C), Tobramicin (TOB), Tetracycline (TE), Tigecycline (TGC), Linezolid (LZD) Fosfomycin (FOS). Microbial strains were classified as sensitive (S), intermediate (I) or resistant (R); all *Vibrio alginolyticus (Va)* isolated from different samples are resistant to P and VA and are sensitive to C.

**Table 3:** Frequency of antibiotic resistance among the bacteria isolated

	**Antiobitic tested**																	
**Samples**	***TEC***	***CN***	***P***	***OX***	***AMC***	***CTX***	***VA***	***SXT***	***RD***	***FOX***	***PRL***	***CIP***	***C***	***TOB***	***TE***	***TGC***	***LZD***	***FOS***
***Ac *** **isolated from *S. scropa***	R	S	R	R	I	S	R	S	S	I	S	S	S	S	S	S	S	S
***Sp *** **isolated from *S. officinalis***	R	S	S	R	S	S	R	S	S	I	S	S	S	I	S	S	S	R
***Sp *** **isolated from *S. officinalis***	R	S	S	R	S	S	R	S	S	I	S	S	S	S	S	S	I	R
***Sp *** **isolated from *G. cobitis***	R	S	R	R	R	I	R	I	S	R	S	S	S	I	I	S	I	S
***Sp *** **isolated from *T. haemastoma***	R	S	R	R	R	I	R	I	S	R	S	S	S	I	I	S	I	S
***Sp *** **isolated from *O. vulgaris***	R	S	S	R	S	S	R	S	S	I	S	S	S	I	S	S	S	R
***Sp *** **isolated from *S. scrofa***	R	S	I	R	S	S	R	S	S	S	S	S	S	S	S	S	S	R
***Sp *** **isolated from *S. scrofa***	R	S	R	R	R	I	R	I	S	R	S	S	S	I	I	S	I	S
***Sp *** **isolated from *S. officinalis***	R	S	R	R	S	S	R	I	I	S	S	S	S	I	S	S	S	S
***Sp *** **isolated from *T. truchurus***	R	I	R	R	S	S	R	S	S	S	S	S	S	I	S	I	S	S
***Ac *** **isolated from *S. scropa***	R	I	R	R	R	I	R	I	S	R	S	S	S	I	S	S	S	S
***Ac *** **isolated from *S. scropa***	R	I	R	R	S	S	R	S	S	R	S	S	S	I	S	I	S	S
***Ac *** **isolated from *D. sargus***	R	S	R	R	S	S	R	I	I	S	S	S	S	I	S	S	S	S
***Ac *** **isolated from *D. sargus***	R	I	R	R	R	I	R	I	S	R	R	S	S	I	S	S	S	S

Teicoplanin (TEC), Cephalexin (CN), Penicillin (P), Oxacillin (OX), Amoxicillin/Clavulanic Acid (AMC), Cefotaxime (CTX), Vancomycin (VA), Sulfamethoxazole (SXT), Rifampicin (RD), Cefoxitin (FOX), Plaritromicin (PRL), Ciprofloxacin (CIP), Chloramphenicol (C), Tobramicin (TOB), Tetracycline (TE), Tigecycline (TGC), Linezolid (LZD) Fosfomycin (FOS). Microbial strains were classified as sensitive (S), intermediate (I) or resistant (R); all microbial strains isolated from different samples are resistant to TEC and VA and are sensitive to C.

Antibiotic resistance profiles among bacterial strains isolates (% resistant strains) were presented in Table 4. As a whole, as supported by statistical analysis, all culturable bacteria were significantly (*P* < 0.001) affected by the presence of the tested antibiotic molecules. In particular, for seven molecules (FOS, FOX, AMC, PRL, TOB, TE and LZD) for the four different bacteria strains isolated a statistically significant (*P* < 0.05) effect (resistance or sensibility) was observed. The other molecules showed any statistically significant effect (*P* > 0.05) among the different isolated microbial strains; in particular, four of them showed very high resistance (mean values: VA: 100%, OX: 98.9%, TEC: 97.7%, P: 85.7%).

**Table 4:** Antibiotic resistance profiles among bacterial strains isolates (% resistant strains)

	**Antibiotics tested**																	
**Microbial strains (n)**	***TEC***	***CN***	***P***	***OX***	***AMC***	***CTX***	***VA***	***SXT***	***RD***	***FOX***	***PRL***	***CIP***	***C***	***TOB***	***TE***	***TGC***	***LZD***	***FOS***
***V. alginolyticus (22)***	90.9	4.5	100	95.5	72.7	13.6	100	4.5	0	4.5	50	0	0	0	9.1	0	0	0
***V. parahaemolyticus (6)***	100	0	100	100	33	0	100	0	0	50	17	0	0	0	0	0	0	0
***S. putrefaciens (7)***	100	0	42.9	100	43	0	100	0	0	43	0	0	0	0	0	0	0	57
***Acromonas spp. (1)***	100	0	100	100	0	0	100	0	0	0	0	0	0	0	0	0	0	0
***Mean of all isolated microbial strains***	97.7	1.1	85.7	98.9	37.2	3.4	100	1.1	0	24.4	16.7	0	0	0	2.3	0	0	14.3

Teicoplanin (TEC), Cephalexin (CN), Penicillin (P), Oxacillin (OX), Amoxicillin/Clavulanic Acid (AMC), Cefotaxime (CTX), Vancomycin (VA), Sulfamethoxazole (SXT), Rifampicin (RD), Cefoxitin (FOX), Plaritromicin (PRL), Ciprofloxacin (CIP), Chloramphenicol (C), Tobramicin (TOB), Tetracycline (TE), Tigecycline (TGC), Linezolid (LZD) Fosfomycin (FOS). All strains are resistant to VA and sensitive to CIP, C, TOB, TGC and LZD.

The study of antibiotic resistance in indigenous water organisms is important, as it might indicate the extent of alteration of water ecosystems by human action. The spread of strains with antibiotic resistance from animal to animal does not meet the minimum barrier in the marine environment and resistance evolves as a consequence of promiscuous exchange and shuffling of genes, genetic platforms, and genetic vectors. Several pollutants in seawater might exert selective activities, as well as ecological damage in water environment, resulting in antibiotic resistance: Baquero *et al*. [18] noted that resistance profiles of aquatic pseudomonads depend on the species composition, but also from the site in which they were isolated, being more antibiotic-resistant along shorelines and in sheltered bays than in the open water, indicating the influence of nonaquatic organisms or pollutants.

### Detection of residues of antibiotics

Residues of antibacterial substances were detected in common octopus, european cuttlefish and red-mouthed rock shell. At the confirmatory analysis only two of the examined drugs were detected. Oxytetracycline and Sulfamethoxazole were quantified with 3.62 μg/kg and 0.48 μg/kg respectively. Levels detected were in compliance with LMR established by UE Reg. 37/2010. The presence of antibiotics might be due to the increased possibility of accumulation in fishery products that, for a period of their life cycle, remain for a long time in the same fishing area. In our study, the sampling area is within a stretch of coastline that spans the mouth of one river and several streams that cross a lot of livestock and agricultural fields.

Studies on residues of pharmacologically active molecules have shown elimination rates in the aquatic environment after the depuration treatment generally between 60 and 90%, for a wide variety of polar drugs [19]. The elimination of drugs in common sewage treatment plants is often incomplete and recent works demonstrates the presence of antimicrobial residues in river waters [20]. Polar antibiotics cannot be eliminated effectively as much of the process of elimination is based on absorption on activated sludge and so ultimately on hydrophobic interactions. Another route of elimination of drugs in the aquatic waste water is connected to the dispersion of manure "contaminated" on the fields as fertilizer through runoff into streams of wastewater and those used for irrigation. It was showed that sulfa drugs, such as sulfadimethoxine, are sufficiently stable in the manure as to maintain a significant residual activity up when the manure is used for fertilizing [21,22] and that some of the metabolites of antibiotics excreted may also be retransformed into the active drug progenitor; such as the glucuronide dicloramphenicol or the N-4-acetyl sulfamethazine converted into the manure in Chloramphenicol and Sulfamethazine respectively [23].

## Conclusions

The risk of antibiotic resistance was considered significantly more serious than the risk associated with the presence of antibiotic residues in food [24]. Results presented in this study provide evidence that seawater fishes collected in some area of Campania Region, especially in marine areas including mouths of streams, were contaminated by residues of antibiotic and antibiotic-resistant bacteria strains and that they might play an important role in the spread of antibiotic-resistance. The resistance of 97.7% of isolated strains against TEC might suggest that the main sources of contamination were hospital discharges.

Future prediction and prevention of antibiotic resistance depends on the research investments in the development of microbial source tracking as well as in the ecology, including water ecology, of antibiotic-resistant microorganisms.

## Methods

### Sampling

Samples were collected always in the same area of the gulf of Salerno (Campania Region, Southern Italy) with the support of the mobile station of the Fish Research Laboratory of the *Department of Veterinary Medicine and Animal Production, University of Naples “Federico II”.* The sampling area was chosen because it is close to sewers conveying hospital wastewater. This study has been reviewed by Ethical Animal Care and Use Committee, University of Naples “Federico II”, Department of Veterinary Medicine and Animal Production and received institutional approval.

The research concerned 56 samples: 33 fish (7 species), 13 cephalopods (2 species) and 10 gasteropods (1 specie) present in the sampling zone in that season; fish species, collected at a depth of 5–7 meters and at a distance of about 50 meters from the coast, were: red scorpionfish (*Scorpaena scrofa,* 6 samples), giant goby (*Gobius cobitis,* 7 samples), atlantic horse mackerel (*Trachurus trachurus,* 4 samples), brown meagre (*Sciaena umbra,* 3 samples), white seabream (*Diplodus sargus,* 6 samples), fathead mullet (*Mugil cephalus,* 5 samples), green wrasse (*Labrus viridis,* 2 samples), common octopus (*Octopus vulgaris,* 7 samples), european cuttlefish (*Sepia officinalis,* 6 samples) and red-mouthed rock shell (*Thais haemastoma,* 10 samples) respectively. Samples after capture were immediately transported on ice to the lab “*Prof.ssa Teresa Antonietta Sarli*” of the *Department of Veterinary Medicine and Animal Production, University of Naples "Federico II”.* An aliquot was subjected to microbiological analysis and the other was frozen at -80°C until analyzed.

### Microbial analysis and antibiogram

All samples were analyzed for the presence of microbial species of the genus *"Vibrio"* according to recognized ISO methods. Briefly all samples were scrubbed and analytical portions (25 g) were aseptically removed and collected in a sterile bag with 225 ml of alkaline saline peptone water (ASPW). According to ISO/TS 21872–1:2007 [25] and ISO/TS 21872–2:2007 [26] indications for fresh products, the samples were homogenized using a stomacher (PBI International, Milan, Italy) at 11000 rev min^-1^ for 3 min and incubated at 37°C [26] and 42°C [25] for 6 h. A further enrichment was performed employing 1 ml of the first enrichment and 9 ml of ASPW. This broth culture was incubated at 37°C and 42°C for 18 h. The enrichment cultures from incubation were plated onto thiosulphate-citrate-bile salt sucrose (TCBS) (Oxoid, Hampshire, UK) agar and incubated at 37°C for 24 h. Typical colonies were transferred into Nutrient Agar plates (Oxoid, Hampshire, UK) added to 5 g/l NaCl to bring it to a final concentration of 1% and incubated at 37°C for 24 h according to ISO/TS method. After incubation at 37°C for 24 h, the isolates were subjected to the Gram stain, the oxidase test using Oxidase Sticks (Oxoid, Hampshire, UK), Triple-Sugar-Iron (TSI) (Oxoid, Hampshire, UK) and biochemical identification with API 20E (bioMérieux, Marcy l’Étoile, France) according to Di Pinto *et al.*[27]. The identification profiles were obtained by the APIweb software (bioMérieux, Marcy l’Étoile, France) according to to the instructions of the manufacturer.

The strains isolated were subjected to the antibiotic resistance test using standard methods. Antibiotic susceptibility was determined by the agar diffusion method according to French national guidelines [28]. Bacterial suspensions prepared in sterile 0.85% saline matching an optical density of 0.5 McFarland standard corresponding to 10^8^ cfu/ml and diluted 1:100 in physiological saline were inoculated by lawn onto Muller-Hinton agar (Difco, Le Pont de Claix, France). Each antibiotic test was run in duplicate on freshly prepared agar plates. After incubation for 24 h at 37°C, organisms were classified as sensitive (S), intermediate (I) or resistant (R) according to the inhibition zone diameter [28].

### Detection of residues of antibiotics

Analysis were performed on fish muscle. Each sample analyzed consisted of a pool of fish and fishery products grouped by species (i.e. sample of red scorpionfish consisted of a pool of six red scorpionfish caught). The detection of antibiotics residues was carried out using the kit “*Premi® Test*” (*Biopharm, Darmstadt, Germany*), as a screening method, according manufacturer's instructions. The kit is based on the growth inhibition of *Bacillus stearothermophilus*, a microorganism sensible to the residues of different antibiotics. This test is able to detect residues of β-lactam antibiotics, cephalosporins, macrolides, tetracyclines, sulfonamides, aminoglycosides, quinolones, amphenicols and polypeptides. The principle on which is based the test is the following: a standard number of spores is embedded in an agar medium with selected nutrients. When *Premi® Test* is heated to 64°C, spores can germinate. If antimicrobial substances are absent, spores germinated producing hydrogen and a clear color change from purple to yellow occurs. When anti-microbial compounds are present above limit of detection, spores will not be able to germinate and there will be no colour change.

Samples positive to *Premi® Test* were analyzed by the mean of HPLC-DAD method suggested by Fernandez-Torres *et al*. [29] for the following compounds (97–99.9% purity, Sigma-Aldrich - USA): Sulfadiazine (SDI), Trimetroprim (TMP), Oxytetracycline (OXT), C and SXT. HPLC-UV method proposed by De Jesùs Valle *et al*. [30], was used for VA detection. Antibiotic selection was made considering drugs commonly used in farms in the Campania region [31]. All reagents used were of analytical grade. Measurements were made with a Jasco (Mary's Court, Easton, MD, USA) liquid chromatograph equipped with UV and diode array (DAD) detector, an injector with a loop of 50 μL, a quaternary pump, a vacuum degasser an a thermostated column compartment. For first method the separation of the analyzed compounds was conducted by means of a Phenomenex C_18_ (150 mm × 4.6 mm I.D., particle size 5 μm) analytical column with a C_18_ (4 mm × 4 mm, particle size 5 μm) guard-column. The mobile phase consisted of a mixture of 0.1% (v/v) formic acid in water pH 2.6 (phase A) and acetonitrile (phase B). A gradient eluition program at 1 mL/m-1 flow rate was used. After a step of 8 min with 99% (phase A) a linear elution gradient to 65% in 25 min was performed. The column effluent was monitored by DAD detector in the range of 200–400 nm. The sample extraction was conducted as follows: After homogenization of sample (2 g of lyophilized tissue + 5 of deionized water) 50 μL of Proteinase-K solution was added; to the mixture, centrifuged for 2.5 hours, 100 μL of formic acid was then added. Finally the samples was treated three times with 5 mL of dichloromethane and the extracts were evaporated under nitrogen. 50 μL of the residue, reconstituted with 1 mL of deionized water, were injected.

For the vancomycin detection method [30], chromatographic separation was carried out by the means of a Nucleosil 120 C18 5 μm column (length, 15 cm; inner diameter, 0.4 cm) using a mixture of 50 mM NH4H2PO4 (pH 4)–acetonitrile (92:8, v/v) as the mobile phase at a flow rate 1 mL/min and a column temperature of 40°C with UV detection at 220 nm. Regarding extraction procedure briefly: a mixture of 500 μg of sample with 20 μL of 60% perchloric acid was vortexed for 30 s, followed by centrifugation at 10,900 rpm, after the supernatant was collected and an aliquot of 50 μL was injected into the chromatographic system.

### Statistical analysis

The χ^2^ test performed with the Epi-Info statistical program (version 6.0; Centers for Diseases Control and Prevention, Atlanta, GA, USA) was used to test the effect of the antibiotics on the bacteria growth and to assess the effect type (resistance or sensibility) of each molecule among the different isolated microbial strain.

## Competing interests

The authors declare that they have no competing interests.

## Authors’ contributions

GS, RM, and SC conceived and designed the experiments; GS and GAM performer the experiments; All authors contributed in the writing of the paper. GO, MLC and AA summarized and analized the data, reviewed and commented the manuscript. All authors read and approved the final manuscript.
